# Chronic cyclic mild heat stress downregulates genes encoding muscle structural components, contributing to reduced breast muscle mass in broiler chickens with minimal impact on meat quality

**DOI:** 10.1093/jas/skaf310

**Published:** 2025-08-31

**Authors:** Ahlem Achour, Moustafa Yehia, Angel Rene Alfonso-Avila, Jean-Michel Allard Prus, Véronique Ouellet, Nabeel Alnahhas

**Affiliations:** Department of Animal Science, Faculty of Agricultural and Food Sciences, Université Laval, Quebec City, QC, G1V 0A6, Canada; Department of Animal Science, Faculty of Agricultural and Food Sciences, Université Laval, Quebec City, QC, G1V 0A6, Canada; Deschambault Research Center in Animal Science (CRSAD), Deschambault, QC, G0A 1S0, Canada; Scott Hatchery, 1798 Rue du Président Kennedy, Scott, QC, G0S 3G0, Canada; Department of Animal Science, Faculty of Agricultural and Food Sciences, Université Laval, Quebec City, QC, G1V 0A6, Canada; Department of Animal Science, Faculty of Agricultural and Food Sciences, Université Laval, Quebec City, QC, G1V 0A6, Canada; Swine and Poultry Infectious Diseases Research Center, Faculty of Veterinary Medicine, Université de Montréal, St-Hyacinthe, QC, J2S 2M2, Canada

**Keywords:** broiler chicken, extracellular matrix, gene expression, hyperthermia, meat quality, reduced yield

## Abstract

Heat stress (**HS**), whether constant or cyclic, poses a significant challenge to the poultry industry, leading to reduced performance, compromised animal welfare, and health issues. Despite its well-known effects, the specific impact of HS on muscle growth and development remains incompletely understood. This study investigated the effects of chronic cyclic mild HS on body weight (**BW**), breast muscle yield (**BMY**), meat quality, glycolytic metabolites, and gene expression in the breast muscles of finishing broilers. A total of 900 Ross 308 chicks (450 males and 450 females) were reared under thermoneutral (**TN**, *n* = 10 pens, 5 pens/sex, 45 birds/pen, 450 birds in total) or HS (*n* = 10 pens, 5 pens/sex, 45 birds/pen, 450 birds in total) conditions (30 °C, 45% RH for 10 h/d from days 28 to 34). On day 35, 2 birds per pen were sampled for meat quality and RNA-Seq analysis. HS reduced BW (*P* = 0.002) and Pectoralis major muscle yield (*P* = 0.02) and tended to decrease BMY (*P* = 0.06). Meat quality traits, glycolytic metabolites, and muscle protein functional properties were not affected (*P* > 0.05), likely due to compensatory feed intake before and after HS exposure. Transcriptomic analysis revealed 301 differentially expressed genes (**DEGs**), including 138 downregulated and 163 upregulated genes under HS (|Log_2_FC| > 1.0, *P* < 0.05). Several downregulated DEGs showed significant positive correlations with BW and muscle mass. Functional enrichment indicated these genes were involved in structural pathways, including extracellular matrix-receptor interaction, cytoskeleton organization in muscle cells, and regulation of the actin cytoskeleton. These pathways are essential for muscle integrity, growth, and repair. Our findings suggest a previously underexplored mechanism by which HS impairs broiler performance through disruption of structural gene networks critical for muscle development.

## Teaser

This study reveals that chronic mild heat stress disrupts key structural genes essential for muscle growth and integrity, highlighting a lesser-known mechanism by which heat stress impairs broiler development.

## Introduction

To meet the growing global demand for poultry meat, the poultry industry relies on intensive genetic selection to develop high-performing broiler strains with faster growth rates, greater feed efficiency, and greater breast meat yield (Zuidhof et al., 2014). These genetic improvements are associated with high metabolic rates and increased heat production (Nascimento et al., 2017). Elevated heat production, insulating plumage, and the absence of sweat glands collectively make modern broilers inefficient in terms of thermoregulation, with a high susceptibility to environmental stressors (Tabler et al., 2020; Malila et al., 2021a). Given the steadily increasing global surface temperatures, the number and frequency of heat waves, heat stress (**HS**) has become a pressing challenge for poultry producers, with forecasts indicating continued economic losses in the coming years (Kumar et al., 2021).

In Canada, surface temperatures are increasing at about double the rate of the global increase (VanderZaag et al., 2024). In Quebec, where the current study was conducted, Ouellet et al. (2021) reported an increased frequency and duration of high Temperature - Humidity Index (**THI**) values in the Eastern and Southwestern parts of the province. Compared with the 1971 - 2000 reference period, the number of days per year with a maximum THI greater than 68 is projected to increase by an average of 18 d in Eastern Quebec and 39 d in Southwestern Quebec during the 2020 to 2049 period. For the 2050 to 2079 period, the projected increases are even larger, with 35 additional days in Eastern Quebec and 63 additional days in Southwestern Quebec.

Many studies have reported that both constant and cyclic HS reduce body weight (**BW**) and breast meat yield (**BMY**) in broilers. For instance, Emami et al. (2021) exposed broiler chickens to a cyclic HS program of 35 °C for 8 h/d from days 29 to 42. They observed a significant decrease in BW under HS compared to thermoneutral (**TN**) conditions at the end of the experiment (3,215 vs. 3,701 g, respectively). Similarly, BMY was significantly lower under HS than TN conditions (29.18% vs. 31.07%). Importantly, their experimental design included a pair-fed group, allowing them to isolate the intrinsic effects of HS from those caused by reduced feed intake. Based on these comparisons, the authors concluded that HS negatively affected weight gain and meat yield independently of the reduction in feed intake under HS conditions. In a different study that also included a pair-fed group, Teyssier et al. (2022) compared cyclic (35 °C for 12 h/d) and constant (35 °C 24 h/d) HS programs of severe intensity applied from days 21 to 41. Compared to the TN thermal condition, both HS programs reduced the yield of the Pectoralis major muscle (19.3% vs 20.89%) and that of the whole breast (23.25% vs 24.80%). These authors also concluded that the detrimental effects of HS cannot be entirely explained by reduced feed intake and that HS per se affected protein and lipid deposition. Muscle protein breakdown under HS conditions can be partly attributed to oxidative damage caused directly by elevated temperatures and to increased corticosterone, accelerating protein breakdown to provide substrates for gluconeogenesis (Ma et al., 2021a, b; Cartoni Mancinelli et al., 2023). In addition to this catabolic effect, HS could also impair growth and muscle development by diverting nutrients and energy from these processes to the immune system to manage the inflammatory processes associated with HS (Zmrhal et al., 2023).

Another mechanism by which HS may reduce muscle growth and development is through the impaired expression of structural proteins, such as those of the extracellular matrix (**ECM**) and their interacting partners. This mechanism remains largely underexplored and is less well understood than the metabolic consequences of HS. In muscle tissue, the ECM is defined as all the secreted molecules extrinsic to the cell, composed of collagens, proteoglycans, and non-collagenous glycoproteins (Velleman, 2020). The ECM is heavily involved in maintaining muscle structural integrity and supporting repair, cell—cell interactions, cell proliferation, differentiation, and fusion during the formation of mature muscle fibers (Velleman, 2020). It thus plays an essential role in muscle tissue growth and regeneration by regulating interactions with the cell surface receptors (Ahmad et al., 2023). Despite its importance for muscle growth and development, very little is known about the impact of HS thermal conditions on the expression of the components of the ECM in broiler breast muscles.

Regarding meat quality, HS can alter final product quality through multiple mechanisms. First, HS can accelerate postmortem acidification in the muscle, leading to a rapid postmortem pH decline, particularly when the carcass temperature is still elevated, which supports continued glycolytic activity and hence the increased accumulation of lactic acid in muscle tissue (Zaboli et al., 2019). This deep decline in postmortem pH is associated with paler meat color as well as greater drip and cooking losses, similar to pale, soft, and exudative (**PSE**)-Like meat (Ziober et al., 2010; Leishman et al., 2021).

It is important to note that the effect of HS on broiler meat quality traits varies depending on the stress program severity (mild, moderate, severe), duration (acute vs chronic), and pattern (cyclic vs constant). Regarding muscle pH, the meta-analysis of Leishman et al. (2021) revealed that acute HS (<24 h) had a greater impact on both the initial (**pHi**) and ultimate pH (**pHu**) compared with chronic HS (>24 h). The study of Zhang et al. (2012) also showed that constant HS (35 °C from 4 to 6 wk of age) induced significantly lower pHi and pHu than cyclic HS (36 °C, 8 h/d from 4 to 6 wk of age). It is well established that technological and sensory quality traits such as color, texture, and water-holding capacity are largely determined by postmortem muscle pH and, more specifically, the rate and ultimate point of pH decline (Alnahhas et al., 2014). Consequently, variations in the postmortem pH lead to deterioration in meat quality, such as increased water loss, abnormally soft texture, and pale color in the case of a high rate of decline (PSE-Like) or greater ultimate point of decline (acid meat). Second, HS influences meat quality through oxidative stress in muscle tissue (Zaboli et al., 2019). In their study, Cartoni Mancinelli et al. (2023) exposed broiler chickens to a constant HS program of mild intensity (30 °C from days 35 to 41) and observed a significant increase in protein oxidation in breast meat samples from stressed birds as compared to those from TN conditions. In a different study, broilers were exposed to an acute HS program of extreme severity (41 °C for 0, 1, 2, 3, and 5 h) followed by the assessment of intramuscular fat peroxidation by measuring breast muscle malondialdehyde concentration at each time point (Wang et al., 2009). These authors found significantly increased malondialdehyde concentrations in samples from broilers exposed to HS for 3 and 5 h when compared to those from TN conditions. The oxidation of muscle proteins and the peroxidation of intramuscular fat are associated with reduced oxidative stability of the final product and a degradation of its quality attributes.

Despite the above-discussed studies, the physiological mechanisms or pathways through which cyclic HS reduces BW and BMY remain unclear. To address this gap, the present study investigates the impact of a chronic cyclic mild HS program, designed to mimic typical local summer conditions outside of peak heat waves, on broiler performance, meat quality, glycolytic metabolites, and gene expression in breast muscles. Unlike previous studies that have primarily focused on metabolism-related pathways, we hypothesize that chronic cyclic HS downregulates the expression of genes encoding muscle structural components, such as those involved in the ECM and actin cytoskeleton, thereby contributing to muscle mass loss and reduced BW, with limited impact on meat quality.

## Materials and Methods

### Ethics statement

This study was conducted at the Deschambault Research Center in Animal Science (Deschambault, Quebec, Canada) in accordance with the guidelines of the Canadian Council on Animal Care and was approved by the Institutional Animal Care and Use Committee of Université Laval (protocol number 2022-1016).

### Experimental design and housing

A total of 900 1-d-old Ross 308 chicks (*n* = 450 males and *n *= 450 females) were acquired from a commercial hatchery (Couvoir Scott, Scott, Quebec, Canada) and placed in an experimental poultry house at the Deschambault Research Center in Animal Science (Deschambault, Quebec, Canada). The house was divided into 2 environmentally controlled rooms: a TN and an HS room. Within each room, 5 randomly chosen floor pens housed males and 5 other randomly chosen pens housed females, with 45 birds per pen (*n* = 450 birds per condition), resulting in a final rearing density of 31 kg/m². The floor pens were bedded with sawdust and equipped with bell drinkers and manually filled circular feeders.

This study aimed to evaluate the impact of HS on traits of interest during the finisher phase, when birds are most susceptible to HS. From days 1 to 27, birds in both environmental rooms of the poultry house were kept under the same environmental conditions corresponding to their requirements. The ambient temperature was maintained at 33 °C during the first week post-hatch, gradually reduced to 22 °C by the end of the third week, and then maintained at 22 °C up to day 27 of the experiment. On day 28, birds placed in the TN room continued to be kept under TN (22 °C, 45% relative humidity) conditions. As for birds placed in the HS room, they were exposed to a cyclical HS program up to the end of the experiment on day 34. The HS program consisted of increasing the ambient temperature to 30 °C while the relative humidity was maintained between 40% and 45% from 6:00 to 16:00. At the end of the daily HS program, the environmental conditions were returned to TN.

Regarding the lighting program, the photoperiod was set to 23 h per day for the first 4 d to allow the chicks to discover their environment and to identify the drinkers and feeders. It was then gradually reduced to 18 h of light and 6 h of darkness (18L:6D) per day. This lighting program was then maintained until the end of the experiment. The photoperiod started at 4:00 and ended at 22:00. During the experiment, birds were fed ad libitum using the starter (AMEn of 2,972.8 kcal/kg and 23.71% of crude protein), grower (AMEn of 3,049.4 kcal/kg and 21.67% of crude protein) and finisher (AMEn of 3,099.4 kcal/kg and 19.5% crude protein) diets described by Yehia et al. (2024).

### Sampling

On day 35, 2 birds per pen were randomly selected from the 45 birds in each pen (*n* = 20 birds per condition, 10 males and 10 females). Random selection was used to avoid sampling bias and ensure that the birds were representative of the pen population. Selected birds were individually weighed and then euthanized by cervical dislocation. Next, the left breast was extracted, and the Pectoralis major and minor muscles were separated. Samples were taken from the cranial part of the Pectoralis major muscle for the evaluation of muscle protein functional properties, glycolytic metabolites (approximately 5 g each), and for RNA extraction (50 mg of muscle tissue/sample). These samples were immediately frozen in liquid nitrogen, transported on dry ice, and stored at −80 °C. Additionally, a sample of approximately 60 g was taken from the midpart of the left Pectoralis major muscle and stored at −20 °C for meat quality analysis. The whole right Pectoralis major muscle was also sampled, weighed (W1), and utilized for the measurement of technological quality traits and breast meat yield.

### Breast meat yield and technological quality traits

Breast meat yield was expressed as a percentage of BW (W1/BW × 100) at slaughter. Meat quality traits were measured after 24 h at 4 °C as described by Sammari et al. (2023). Briefly, the pHu was measured using a portable pH meter (Ross, Orion Star A221, Thermo Scientific, Beverly, CA, USA) equipped with an Orion Kniphe electrode (Thermo Fisher, Nepean, ON, Canada) and a temperature compensation probe (928007MD, Micro ATC probes, Maryland, USA) by direct insertion in the thickest (cranial) part of the Pectoralis major muscle from the dorsal (bone side) surface. As for meat color, it was measured on the same spot as the pH using a Chromameter (Chromameter CR-400, Minolta Ltd., Osaka, Japan) equipped with a conical open port and an 8 mm aperture, a diffuse illumination with 0° viewing angle geometry, and a D65 light source according to the CIE trichromatic color system (L*: lightness, a*: redness, and b*: yellowness). Measurements of pHu and color were performed in a cold room (4 °C) under standardized artificial lighting conditions. For both traits, measurements were conducted approximately 30 min after samples were removed from storage. Breast fillets were then placed back in their bags, kept at 4 °C for 72 h then they were gently wiped with an absorbent paper, weighed again (W2), and the drip loss (**DL**) was calculated as the difference between W1 and W2 expressed as a percentage of W1. Next, a sample of approximately 60 g was taken from the midpart of each breast fillet, placed in a plastic bag (Whirl-Pak bag, Nasco Whirl-Pak^®^, USA), and cooked in a water bath at 85 °C until an internal temperature of 76 °C was reached. The endpoint temperature was monitored by inserting a digital probe into the core of the meat sample, which provided continuous real-time temperature monitoring. The samples were then cooled down in an ice water bath for 10 min, taken out of their bags, wiped gently with absorbent paper, and weighed again. The cooking loss (**CL**) was expressed as a percentage of sample’s initial weight before cooking. Cooked meat samples were then cut into strips (1 cm × 1 cm × 3 cm) parallel to the direction of the muscle fibers to evaluate their shear force using a texture analyzer (ZwickiLine, Zwick/Roell, Germany) equipped with a Warner-Bratzler blade moving at a crosshead speed of 200 mm/min. This test was performed in triplicate and the average of the maximum force (N/cm^2^) required to shear the replicates was reported.

After 30 d at −20 °C, samples from the left Pectoralis major muscle were thawed overnight at 4 °C, and freezing-thawing loss was calculated as the difference in sample weight before freezing and after thawing and then expressed as a percentage relative to the weight before freezing. Subsequently, thawed samples were marinated as described by Mudalal et al. (2015). Briefly, breast meat samples were marinated with 150 mL of solution per kg of meat, containing 7.6% sodium chloride and 2.3% triphosphate. Marination was performed using a small-scale vacuum tumbler (Promarks TM-150, Canada) for 46 minutes under vacuum (−0.95 bar), at a total of 800 revolutions. The tumbling process consisted of three working cycles of 13 minutes each, separated by 2 pause cycles of 3 minutes each. Marinade uptake was evaluated as the difference in weight of the meat sample before and after marination. To determine CL after freezing, thawing, and marination (**CL2**), meat samples were cooked in an oven using the hot air mode (Rational, SelfCookingCenter® 5 Senses 102E) until an internal sample temperature of 76 °C. After cooking, samples were left to cool down at room temperature, weighed again, and the CL was expressed as a percentage of the sample weight before cooking. Meullenet-Owens razor shear force was measured on cooked meat samples using a texture analyzer (Model TAXT Plus, Texture Technologies Corp., Scarsdale, NY) set at crosshead speed of 5 mm/s and a shear depth of 20 mm.

### Functional properties of breast muscle proteins

The solubility, emulsion activity index (**EAI**), and emulsion stability index (**ESI**) were measured as described by Benahmed et al. (2023). Briefly, duplicate 1 g samples were homogenized in 20 mL ice-cold 25 mM potassium phosphate buffer (pH 7.2). The homogenates were centrifuged at 15,300 × g for 15 min at 4 °C, and the supernatant was carefully transferred to a new tube through a Whatman N°1 filter paper. The solubility of the sarcoplasmic fraction was determined by measuring the protein concentration of this supernatant using a BCA assay (Pierce™ BCA Protein Assay Kit, Thermo Fisher Scientific) with a BSA standard curve. The solubility of the myofibrillar fraction was similarly determined after homogenizing the remaining pellet in 20 mL ice-cold buffer (0.55 M KI, 50 mM potassium phosphate at pH 7.2), centrifuging at 15,300 × g for 15 min at 4 °C, and pouring the supernatant carefully into a new tube through a Whatman N°1 filter paper (Bowker and Zhuang, 2016).

After determining the protein concentration of the sarcoplasmic and myofibrillar fractions as described above, it was adjusted to 1.5 mg/mL in both fractions, mixed with corn oil in a 3:1 ratio (volume/volume), and homogenized at 12,000 rpm for 1 min using an Ultraturrax (IKA, Wilmington, NC, USA). Aliquots of the emulsions (35 µL) were diluted to 3.5 mL in 0.1% SDS buffer in quadruplicate. Immediately after dilution, the turbidity was read at 500 nm using a spectrophotometer (Genesys 10S UV-Vis, Thermo Scientific). As described by Benahmed et al. (2023), the EAI was then calculated as: EAI = 2.33 × A0, where A0 is the absorbance of the emulsion. To determine the ESI, the absorbance of the emulsions was read a second time after settling at room temperature for 20 min without agitation. The ESI was then calculated as: ESI = 10 × [A0/(A0 − A20)], where A0 is defined as before and A20 is the absorbance measured 20 min after A0.

### Muscle glycolytic metabolites

Glycolytic metabolites (lactate, glucose, and glucose-6-phosphate) of the Pectoralis major muscle were analyzed (*n* = 20 samples/treatment group, 10 per sex) in duplicates according to Baldi et al. (2020). Briefly, glucose and glucose-6-P were measured through a coupled reaction in which hexokinase (Sigma, 11426362001, Canada) phosphorylated glucose to glucose-6-P, and glucose-6-P dehydrogenase (Sigma, G5760, Canada) catalyzed the oxidation of glucose-6-P to 6-phosphogluconate, reducing NAD^+^ to NADH. The change in absorbance at 340 nm was proportional to the glucose and glucose-6-P concentrations, calculated by comparing sample values to standards. Lactate concentration was measured by the enzymatic conversion of lactate to pyruvate using lactate dehydrogenase (Sigma, L7525, Canada), with NAD^+^ as a cofactor, and the resulting NADH production was measured at 340 nm. All concentrations were normalized to sample weight and expressed as µmol/g of fresh tissue.

### RNA extraction and sequencing

For RNA-Seq, 6 birds per treatment (3 males and 3 females) were randomly selected from the initial pool of 20 birds sampled per group (see section ‘Sampling’), with birds originating from different pens to ensure representative sampling across pens and sexes. The number of replicates (*n* = 6 samples/group) is consistent with commonly used RNA-Seq designs in poultry transcriptomics and provides sufficient power to detect biologically relevant gene expression changes when combined with appropriate statistical methods such as DESeq2, which accounts for dispersion estimates in small sample sizes (Love et al., 2014).

The RNA was isolated from the samples using the TRIzol™ Reagent (Invitrogen, Canada) following the manufacturer's recommendations. Then, the RNA was quantified using spectrophotometry (Nanodrop, Thermo Scientific, Canada). A quality control was also performed using a Bioanalyzer (RNA 6000 Nano chip, Agilent, Canada), which confirmed that the quality of the isolated RNA was acceptable for use throughout the rest of the protocol (average RNA integrity number of 8.70 and 8.75 in the HS and TN thermal environments, respectively). Next, the sequencing libraries were prepared using the NEBNext Ultra II Directional RNA library preparation kit for Illumina, which includes Poly(A) mRNA magnetic isolation module (New England Biolabs, Canada) using 1 µg of RNA as starting material. The quality control of the sequencing libraries was performed using Bioanalyzer with High sensitivity DNA chip (Agilent, Canada) after quantitation using Quant-iT™ dsDNA assay kit (Invitrogen, Canada). Finally, libraries were sequenced using an AVITI™ (Element Biosciences, Canada) sequencer with the Cloudbreak Freestyle SE150 kit (Element Biosciences, Canada) at the Institut de Biologie Intégrative et des Systèmes (IBIS, Université Laval, Quebec, Canada).

### Bioinformatics

The software fastp version 0.24.0 (Chen et al., 2018) was used with default parameters to evaluate the quality of reads, remove low-quality reads, and trim adapter sequences from raw fastq files. Then, transcript quantification was performed on the trimmed fastq files using the *Salmon* pseudo-aligner version 1.10.2 (Patro et al., 2017) with default parameters according to the transcriptome of version 7.0 of the Ensembl chicken genome (GRCg7b).

We then used the web-based software Express Analyst (Ewald et al., 2023) to conduct the differential gene expression (**DGE**) analysis. The DGE analysis was conducted using the DESeq2 method (Love et al., 2014) as implemented in Express Analyst. The primary factor in the analysis was the thermal environment, with the following contrast: HS versus TN. *P*-values were corrected to control the false discovery rate using Benjamini and Hochberg’s method. Because, as mentioned before, each treatment group included an equal number of males and females, sex was not included as a covariate in the statistical DGE model. This balanced allocation eliminates confounding between sex and treatment, ensuring an unbiased estimate of the thermal treatment effect while preserving degrees of freedom.

The identified genes with *P*-value < 0.05 and |Log_2_(fold change)| > 1.0 were considered differentially expressed (**DEGs**), and a volcano plot was created with the Enhanced Volcano R package to present the DEGs. Finally, we used the functional annotation tool in DAVID (Dennis et al., 2003) to identify KEGG pathways to which DEGs were contributing.

### Statistical analysis

A linear mixed effects model was fitted to the performance and meat quality data as implemented in the lmerTest package of R (Kuznetsova et al., 2017). The model included the effect of the pen intra-room as a random effect to account for potential random variation in the environment between pens, while the thermal environment, the sex, and the thermal environment-by-sex interaction were included in the model as fixed effects. Model assumptions, including the normality of residuals and homoscedasticity, were verified by the visual inspection of the residuals’ plots using the plot method in R. Comparisons between treatment groups were performed using the Tukey method as implemented in the emmeans package of R, and results were presented as least squares means ± their standard errors. Differences between means were declared significant at *P* ≤ 0.05 and tendency was declared at 0.05 < *P* ≤ 0.10.

Gene expression—phenotype correlations (*n* = 12 birds, 6 per treatment group) were calculated using the rcorr function from the Hmisc package of R. As we used the normalized counts of transcripts (log of counts per million or CPM) to compute these correlations, we used the Pearson method as implemented in the rcorr function. Statistically significant correlations were presented as heatmaps that were created using the pheatmap package of R.

## Results and Discussion

### BW and breast meat yield

The statistical analysis did not reveal any significant interaction between the environmental condition and sex for the measured performance traits (Table 1). Nevertheless, the application of the HS program led to a significant decrease in both BW (−8.15%), Pectoralis major muscle yield (−6.52%), and tended to decrease whole breast yield (−4.78%). As for the Pectoralis minor muscle yield, it remained unaffected by HS.

**Table 1.** Effect of environmental condition, sex, and their interaction on body weight, feed intake, and breast meat yield at day 34

Trait^1^	Condition^2^			Sex			*P*-value^3^		
	TN	HS	SEM	Males	Females	SEM	C	S	I
BW, g	2,832.0	2,601.0	48.0	2,933.0	2,500.0	51.40	0.002	< 0.001	0.19
FI^4^, g/bird	1,182.0	1,049.0	28.5	1,227.0	1,004.0	28.5	< 0.01	< 0.001	0.44
FCR	2.090	2.690	0.16	2.45	2.33	4.08	< 0.01	< 0.001	0.44
BMY, %	20.9	19.9	0.35	20.60	20.30	0.36	0.06	0.53	0.83
P. major, %	18.4	17.2	0.28	18.10	17.50	0.29	0.02	0.14	0.66
P. minor, %	2.56	2.68	0.11	2.45	2.79	0.11	0.43	0.04	0.78

^1^BW, Body weight; BMY, beast meat yield; P. major, yield of the Pectoralis major muscle; P. minor, yield of the Pectoralis minor muscle (*n* = 20 birds/condition, 10 birds/sex/condition).

^2^TN, Thermoneutral (22 °C, 45% RH), HS, heat stress (30 ^°^C, 45% RH, 10h/d from days 28 to 34).

^3^C, *P*-value of the environmental condition; S, *P*-value of the effect of sex; I, *P*-value of the sex-by-environmental condition interaction.

^4^Feed intake (average of 10 pens/condition, 5 pens/sex/condition) during the stress period (days 28 to 34).

Losses in BW and muscle mass in broilers exposed to HS can be attributed to both feed intake-related factors and HS-related factors (Teyssier et al., 2023). It is well established that broilers consume less feed under HS conditions, an adaptive response permitting birds to reduce their basal metabolic rate and heat production to limit further increases in body temperature (Andretta et al., 2021). In our previous study (Yehia et al., 2025), from which the birds used in the current study were sampled, broilers exposed to the cyclic HS program had lower feed intake and greater feed conversion ratio compared with TN birds during the stress period between days 28 and 34 (Table 1). Despite this adaptive response, HS birds exhibited significantly greater rectal temperatures (41.28 ± 0.04 °C vs 39.78 ± 0.04 °C, *P* < 0.001), suggesting that thermoregulatory mechanisms did not fully offset heat stress-induced metabolic disruptions. Similar patterns of reduced feed intake (160 vs 196 g.bird^-1^.day^-1^, *P* < 0.0001), increased feed conversion ratio (1.99 vs 1.85, *P* = 0.006), and elevated facial (skin) temperature were observed in another study (Yehia et al., 2024) that applied a more severe cyclic HS program (34 °C, 55% to 60% RH), which also suggested that thermoregulatory mechanisms were not successful in offsetting HS-induced metabolic disruptions. It could thus be argued that under cyclic HS, reduced BW and meat yield are partly a consequence of reduced feed intake and deteriorated feed efficiency (Emami et al., 2021). Additionally, HS is known to compromise intestinal integrity, compromising the metabolic function of enterocytes and facilitating bacterial infections (Tabler et al., 2020; Emami et al., 2021), which negatively impact digestion and nutrient absorption, leading to further decreases in BW and BMY (Teyssier et al., 2022). Compromised nutrient absorption, coupled with increased metabolic demands for thermoregulation and immune function, may intensify muscle mass loss under HS conditions. In their study using pair-feeding (birds under TN conditions fed the same quantity consumed by birds under HS conditions), Teyssier et al. (2022) reported that 19% of the loss in BW and 36% of the loss in BMY were directly attributed to the effect of HS per se, and not to reduced feed intake. These authors highlighted the potential of HS to significantly reduce protein accretion in broiler chickens, which aligns with a previous study showing that chronic constant HS (32 °C for 1 wk in the finisher phase) increased muscle protein breakdown through elevated circulating cortisone levels, leading to breast muscle atrophy (Ma et al., 2021b). The breakdown of muscle proteins releases amino acids that can be utilized via gluconeogenesis to produce energy, a process that, under HS, could further contribute to the loss of muscle mass (Ma et al., 2021b).

### Breast meat quality

The effect of sex-by-thermal condition interaction was not significant for any of the measured meat quality traits (Table 2). Moreover, meat quality traits were not influenced by the effect of the environmental condition. These findings are in agreement with findings from the study of Malila et al. (2021a). These authors exposed commercial broilers to a cyclic HS program of severe intensity (35 °C for 6 h/d for 3 wk) and showed that breast meat quality traits, including pHu, DL, CL, and a*, did not differ between the HS and TN thermal conditions. Our findings also align with those of Teyssier et al. (2022), who compared a chronic cyclic (35 °C, 12 h/d from 20 to 41 d) and a constant (35 °C, 24 h/d from 20 to 41 d) HS program of severe intensity and reported a decrease in the pHu only under the constant HS program, similar to findings from other studies (Orlowski et al., 2020; Greene et al., 2021).

**Table 2.** Effect of environmental condition, sex, and their interaction on breast meat quality traits (*n* = 20 birds/group, 10 birds/sex)

Traits^1^	Condition^2^			Sex			P-Value^3^		
	TN	HS	SEM	Males	Females	SEM	C	S	I
pHu	5.88	5.78	0.02	5.89	5.86	0.02	0.64	0.11	0.48
L*	55.90	57.10	0.78	56.00	56.90	0.74	0.25	0.43	0.58
a*	8.77	7.43	0.76	8.91	7.29	0.73	0.21	0.13	0.49
b*	13.60	14.00	0.70	14.00	13.60	0.67	0.64	0.69	0.89
DL, %	4.92	4.60	0.49	4.67	4.84	0.47	0.62	0.79	0.67
CL, %	16.40	16.30	0.45	16.80	16.00	0.42	0.94	0.19	0.75
WBSF, N/cm^2^	19.70	21.40	1.02	19.60	21.50	0.96	0.22	0.18	0.40
Freeze-Thaw, %	15.00	14.80	1.34	13.70	16.10	1.26	0.90	0.18	0.77
Marinade uptake, %	6.91	7.46	0.38	6.78	7.59	0.38	0.32	0.14	0.08
CL2, %	14.40	14.70	0.33	14.80	14.40	0.34	0.51	0.41	0.81
MORS, N	3.08	3.36	0.17	2.99	3.46	0.17	0.26	0.06	0.37

^1^pHu, Ultimate pH; L*, lightness; a*, redness; b*, yellowness; DL, drip loss; CL, cooking loss; WBSF, Warner-Bratzler shear force; Freeze-Thaw, loss after one cycle of freezing and thawing; CL2, cooking loss after thawing, marination and cooking; MORS, Meullenet-Owens razor shear.

^2^TN, Thermoneutral (22 °C, 45% RH); HS, heat stress (30 ^°^C, 45% RH, 10h/d from day 28 to 34).

^3^C, *P*-value of the environmental condition; S, *P*-value of the effect of sex; I, *P*-value of the sex-by-environmental condition interaction.

The pHu of the Pectoralis major muscle is the major determinant of all other breast meat quality traits (Alnahhas et al., 2014, 2015; Zaboli et al., 2019). This technological quality parameter depends almost entirely on the glycolytic reserves present in the muscle at slaughter (Le Bihan-Duval et al., 2008). As mentioned before, when broilers are exposed to high ambient temperatures, feed intake is reduced, which disrupts muscle glucose homeostasis (Kwakye et al., 2023), leading to an increased mobilization of muscle glycogen reserves to provide the muscle with ATP via anaerobic glycolysis. Under the anaerobic conditions of the Pectoralis major muscle, the pyruvate produced by glycolysis is converted to lactic acid. This accumulation of muscle lactic acid and hydrogen ions (H^+^) is responsible for the postmortem decline of muscle pH in birds exposed to HS (Zaboli et al., 2019). When birds are slaughtered shortly after HS exposure, the combination of accumulated glycolytic metabolites, low pH, and high carcass (i.e., muscle) temperature leads to accelerated protein denaturation and subsequent deterioration of quality traits, including color parameters, texture, and water-holding capacity (Zhang et al., 2019). In the present study, we did not observe any significant differences between HS and TN thermal conditions in terms of glycolytic metabolites (Table 3) or in terms of the denaturation of the myofibrillar and sarcoplasmic fractions of muscle proteins as measured by their solubility (Table 4). In fact, except for the ESI of the sarcoplasmic fraction, which was significantly lower under HS than under TN, the functional properties of muscle proteins remained unchanged in our study (Table 4). The lack of differences in glycolytic metabolites thus explains the absence of differences in the pHu and other meat quality traits that we investigated. Another factor that must be considered is that in our study, the HS program (from 6:00 to 16:00) completely overlapped with the lighting program (from 4:00 to 22:00). This allowed birds in our study 2 h in the early morning and 6 h in the evening to consume feed and compensate for the reduced feed intake during the stress period, although they consumed less feed overall compared to TN group (Yehia et al., 2025). Additionally, the possibility that the absence of significant differences between HS and TN conditions for certain parameters was due to the mild nature of the cyclic HS program used in this study cannot be fully excluded. Moreover, in the present study, the application of HS on the last day of the stress program ended in the afternoon (16:00), while the processing of the birds took place in the morning (9:00) of the next day. We therefore hypothesized that compensatory short-term feed consumption before and after the stress cycle could have allowed the birds to reconstitute the glycolytic reserves of their breast muscles, leading to the reported lack of differences in the glycolytic metabolites (Table 3) and hence the subsequent lack of differences in meat quality attributes. Nevertheless, the short-term feed consumption was not enough for the birds to recover from the overall loss of BW and meat yield observed at the end of the stress program (Table 1). The lasting effects on these two traits may reflect both reduced feed intake during the stress program and intrinsic effects of HS. Pair-feeding experiments would help clarify the relative contributions of these factors. Overall, chronic mild cyclic HS applied for 10 h/d seems to have a slight to no effect on meat quality traits of finishing broiler chickens (days 29 to 34).

**Table 3.** Effect of environmental condition, sex, and their interaction on glycolytic metabolites in the Pectoralis major muscle at death (*n* = 20 birds/group, 10 birds/sex)

Trait	Condition^1^			Sex			*P*-value^2^		
	TN	HS	SEM	Males	Females	SEM	C	S	I
Lactate, µmol/g	56.90	66.90	4.66	60.80	63.00	4.42	0.13	0.72	0.37
Glucose, µmol/g	10.70	10.40	1.33	7.06	14.07	1.26	0.88	0.00	0.75
Glucose-6-P, µmol/g	2.61	2.40	0.44	1.69	1.32	0.42	0.73	0.01	0.28

^1^TN, Thermoneutral (22 °C, 45% RH); HS, heat stress (30 ^°^C, 45% RH, 10 h/d from days 28 to 34).

^2^C, *P*-value of the environmental condition; S, *P*-value of the effect of sex; I, *P*-value of the sex-by-environmental condition interaction.

**Table 4.** Effect of environmental condition, sex, and their interaction on the functional properties of protein fractions of the Pectoralis major muscle (*n* = 20 birds/group, 10 birds/sex)

Trait^1^	Condition^2^			Sex			*P*-value^3^		
	TN	HS	SEM	Males	Females	SEM	C	S	I
	**Myofibrillar proteins**								
Solubility, mg/g	104.0	114.0	4.14	107.0	111.0	4.14	0.08	0.53	0.01
EAI	2.12	2.00	0.07	2.08	2.04	0.07	0.25	0.67	0.57
ESI	35.60	35.50	0.87	35.70	35.40	0.87	0.91	0.78	0.40
	**Sarcoplasmic proteins**								
Solubility, mg/g	94.2	94.0	3.88	91.0	97.1	3.88	0.97	0.28	0.22
EAI	1.4	1.2	0.07	1.4	1.3	0.07	0.09	0.46	0.29
ESI	23.1	21.2	0.53	21.6	22.7	0.53	0.01	0.12	0.25

^1^EAI, emulsion activity index; ESI, emulsion stability index.

^2^TN, Thermoneutral (22 °C, 45% RH); HS, heat stress (30 ^°^C, 45% RH, 10 h/d from days 28 to 34).

^3^C, *P*-value of the environmental condition; S, *P*-value of the effect of sex; I, *P*-value of the sex-by-environmental condition interaction.

Although the primary focus of the present study was the effect of thermal condition on growth and meat quality, we also observed a significant effect of sex on muscle glycolytic metabolites (Table 3). Regardless of thermal condition, female birds exhibited greater free glucose (*P* < 0.001) and slightly lower glucose-6-P (*P* = 0.01) concentrations compared with males. These results are consistent with previous reports indicating that female broilers have greater glycolytic potential in the Pectoralis major muscle than males (Dadgar et al., 2011). While the mechanisms underlying these sex-related differences were not investigated here, they may reflect inherent differences in muscle metabolism and merit further investigation.

### Muscle transcriptome

**Figure 1.**
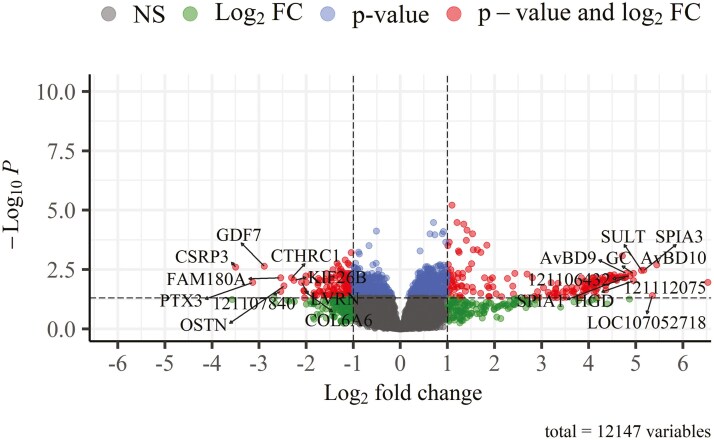
Volcano plot of significantly differentially expressed genes in the Pectoralis major muscle of the heat stress group (30 °C, 40% to 45% relative humidity) relative to the thermoneutral group (22 °C, 45 % relative humidity). Annotated genes are the top 10 most upregulated and downregulated genes. NS, nonsignificant genes (in gray); Log_2_FC, genes with |log_2_(FC)| > 1.0 (in green), *P*-value: genes with *P* < 0.05 (in blue), *P*-value and log_2_FC: genes with |log_2_(FC)| > 1.0 and *P* < 0.05 (in red).

The number of reads and the mapping rate per sample are presented in Table 5. The DGE analysis of samples from the Pectoralis major muscle revealed the presence of 301 significantly (|log_2_(FC)| > 1.0 and *P* < 0.05) differentially expressed genes between the HS and TN thermal conditions (Figure 1). Of these genes, 163 were upregulated and 138 were downregulated under HS relative to TN thermal conditions (Supplementary Table S1).

**Table 5.** Summary statistics of transcriptomic data

Sample	Condition^1^	Reads (M^2^)	Mapping, %
1	HS	26.43	75.90
2	HS	31.81	76.90
3	HS	28.24	76.80
4	HS	29.42	76.45
5	HS	27.00	76.80
6	HS	24.57	78.40
7	TN	31.52	77.45
8	TN	22.91	79.16
9	TN	25.42	80.67
10	TN	25.11	77.12
11	TN	34.66	79.24
12	TN	41.43	79.82

^1^TN, Thermoneutral (22 °C, 45% RH); HS, heat stress (30 ^°^C, 45% RH, 10 h/d from days 28 to 34).

^2^Number of reads expressed in millions.

The results of the pathway enrichment analysis of differentially expressed genes are presented in Supplementary Table S2. For the purposes of the current study, we will only focus on muscle structure-related and ECM-related pathways (Table 6), as there is a gap in the available literature regarding the impact of HS on these pathways in relation to muscle mass loss. The reader is referred to previous studies that provided in-depth discussions of changes in metabolism-related pathways under HS (Zahoor et al., 2017; Wu et al., 2020, 2024; Malila et al., 2022; Li et al., 2024). As can be seen in Supplementary Table S2 and in Table 6, the false discovery rate-corrected *P*-values for the enrichment analysis were not statistically significant, as we only used the DEGs (*n* = 301 genes) against a background consisting of the entire list of genes (*n* = 12,147 genes) in the analysis. The purpose of this analysis was to place DEGs in the pathways they contribute to.

**Table 6.** KEGG pathway enrichment analysis of differentially expressed genes in the Pectoralis major muscle of the heat stress group (30 ^°^C, 45% RH, 10 h/d from days 28 to 34) relative to the thermoneutral group (22 ^°^C, 45% RH)

Pathway	Count	%	FE^1^	P	FDR^2^	Genes^3^
Regulation of actin cytoskeleton	11.00	3.67	2.43	0.01	0.41	FGF7, ITGA8, MYLK2, F2, KNG1, C8B, C7, C6, C8A, MYL10, ITGB8
ECM^4^-receptor interaction	6.00	2.00	3.28	0.03	0.41	ITGA8, COMP, THBS2, VTN, COL6A6, ITGB8
Cytoskeleton in muscle cells	10.00	3.33	2.18	0.04	0.41	CSRP3, ITGA8, TRIM63, COMP, THBS2, MYOZ2, FBLN1, VCAN, COL6A6, ITGB8

^1^Fold enrichment.

^2^False discovery rate-corrected *P*-value.

^3^ *ITGA8* and *ITGB8*, Integrin subunit alpha and beta 8; *COL6A6*, Collagen type VI alpha-6 chain; *VTN*, Vitronectin; *THBS2*, Thrombospondin 2; *COMP*, Cartilage oligomeric matrix protein; *VCAN*, Versican; *FBLN1*, Fibulin 1; *MYOZ2*, Myozenin 2; *TRIM63*, Tripartite motif containing 63; *CSRP3*, Cysteine and glycine rich protein 3; *C6*, *C7*, *C8A*, *C8B*, complement components 6, 7, 8A, and 8B; *MYLK2*, Myosin light chain kinase 2; *MYL10*, Myosin light chain 10; *KNG1*, Kininogen 1; *F2*, Coagulation factor II; *FGF7*, Fibroblast growth factor 7.

^4^ECM, Extracellular matrix.

#### The extracellular matrix (ECM)-receptor interaction pathway (KEGG: gga04512)

**Figure 2.**
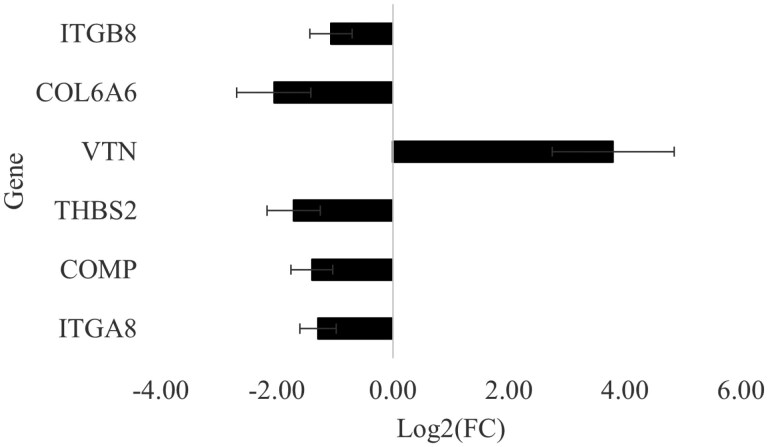
Log_2_(fold change) ± SE of significantly differentially expressed genes classed in the extracellular matrix (ECM)-interaction pathway. *ITGA8* and *ITGB8*, Integrin subunit alpha and beta 8; *COL6A6*, Collagen type VI alpha-6 chain; *VTN*, Vitronectin; *THBS2*, Thrombospondin 2; *COMP*, Cartilage oligomeric matrix protein.

Six of the DEGs were contributing to the ECM-receptor interaction pathway as shown in Figure 2. As can be seen in this figure, except for the *VTN* gene, all these genes were significantly downregulated in the breast muscle of birds kept under the HS thermal condition. The ECM is defined as all the secreted molecules extrinsic to the cell, composed of collagens, proteoglycans, and non-collagenous glycoproteins (Velleman, 2020). It plays an essential role in muscle tissue regeneration by regulating cell proliferation, migration, and differentiation *via* interactions with the cell surface receptors (Ahmad et al., 2023). The downregulation of these genes under HS reveals that HS impairs muscle development and repair by specifically influencing genes in this pathway.

Of the proteins encoded by these genes, integrins (ITG), a group of transmembrane glycoproteins consisting of an α (ITGA, subunit alpha) and β (ITGB, subunit beta) chains that are non-covalently associated, constitute the major family of cell surface receptors responsible for mediating both cell–cell and cell–matrix interactions (Mayer, 2003; Boppart and Mahmassani, 2019). Studies on broiler chickens showed that *ITGA8* was particularly overexpressed in hypertrophic muscles of fast-growing strains affected by breast muscle myopathies, demonstrating the impact of the overexpression of this gene in the increased muscle growth and presence of connective tissue, a hallmark characteristic of myopathies such as Wooden breast (Malila et al., 2021b).

The Cartilage Oligomeric Matrix Protein (*COMP*) gene encodes an ECM glycoprotein, also known as Thrombospondin-5, that contributes to muscle development. In broiler breast muscle, *COMP* knockdown has been shown to reduce satellite cell proliferation during the later stages of myogenesis (Velleman et al., 2021), which impairs muscle growth and hinders its repair when damaged.

The *THBS2* gene also encodes a glycoprotein of the ECM called Thrombospondin-2 that is involved in multiple interactions with cell surface receptors, ECM components, growth factors, enzymes, and calcium binding (Calabro et al., 2014). Similar to *ITGA8*, studies on broiler breast muscles revealed that this gene was upregulated in hypertrophic muscles exhibiting myopathies and was found to exert a pro-fibrotic effect (Brothers et al., 2019; Che et al., 2024), highlighting its role in muscle structure.

The Collagen type VI Alpha-6 chain (*COL6A6*) encoding gene was also downregulated in our study under the effect of HS. In broiler breast muscles, collagen types I and III are the most abundant (Velleman, 2020). Hence, the implications of the downregulation of this gene for muscle structure are unclear. In human skeletal muscles, the downregulation of the alpha-6 chain of collagen type VI was associated with muscle dystrophy (Tagliavini et al., 2014). It is thus plausible that its downregulation by HS could have contributed to the loss of breast muscle mass in birds kept under HS conditions.

The only gene that was upregulated in the ECM-receptor interaction pathway in our study was the Vitronectin (*VTN*) encoding gene. Vitronectin is another ECM protein that is also involved in cell adhesion, migration, and differentiation, and is observed in areas of tissue injury and necrosis (Seiffert, 1997). Consequently, the upregulation of *VTN* in the Pectoralis major muscle under HS thermal conditions could be a response to HS-induced muscle tissue inflammation and adipose infiltration (Malila et al., 2022).

**Figure 3.**
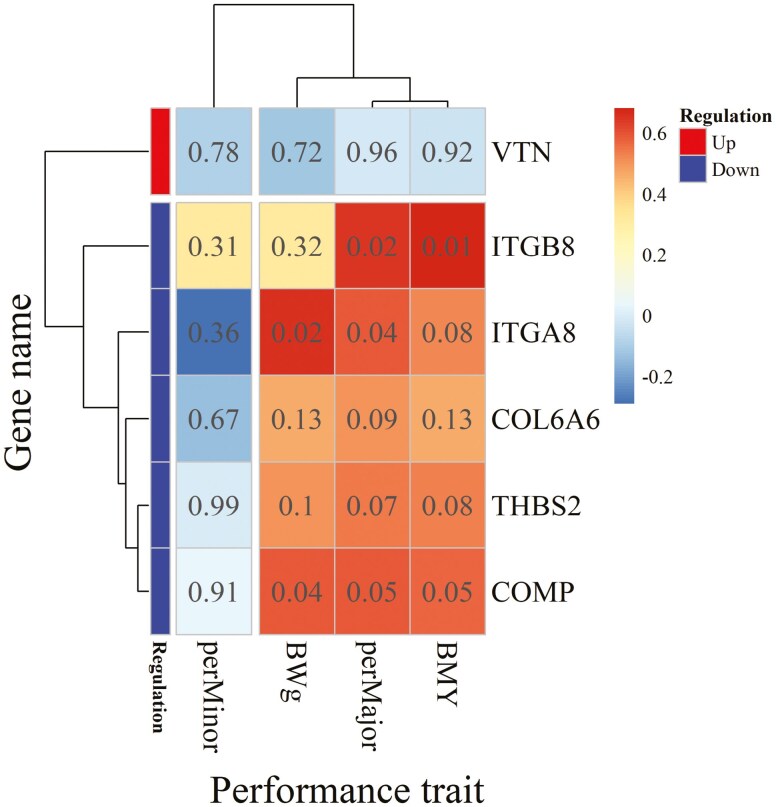
Heatmap of the correlations of significantly differentially expressed genes classed in the extracellular matrix (ECM)-interaction pathway with body weight expressed in grams (BWg), yield of the whole breast (BMY), of the Pectoralis major (perMajor), and Pectoralis minor (perMinor) muscle. *ITGA8* and *ITGB8*, Integrin subunit alpha and beta 8; *COL6A6*, Collagen type VI alpha-6 chain; *VTN*, Vitronectin; *THBS2*, Thrombospondin 2; *COMP*, Cartilage oligomeric matrix protein. The color scale indicates the order of magnitude of the correlation coefficients, and numbers displayed in the cells of the heatmap are *P*-values of corresponding correlation coefficients.

Given their functional roles in muscle structure and growth, we considered that the downregulation of several DEGs in the ECM-receptor interaction pathway might contribute to the lower yield of the Pectoralis major muscle under HS conditions compared with TN conditions. To explore this possibility, we examined correlations (*n* = 12 birds, 6 birds per group) between these DEGs and performance traits including BW, BMY, P. major yield, and P. minor yield (Figure 3). BMY was positively correlated with *ITGB8* and *COMP* expression and showed a tendency to correlate with *ITGA8* and *THBS2*. Similarly, P. major yield was positively correlated with *ITGA8*, *ITGB8*, *COMP*, and tended to correlate with *THBS2* and *COL6A6*. As expected, BW, which reflects overall muscle growth, was positively correlated with *ITGA8* and *COMP*. In contrast, P. minor yield did not correlate with any of the DEGs in this pathway. Overall, these positive correlations are consistent with the hypothesis that HS may reduce muscle growth and development through the downregulation of genes encoding ECM components. Nevertheless, these findings will need to be validated in future studies, including a pair-fed group and using a larger sample size.

#### Cytoskeleton in muscle cell pathway (KEGG: gga04820)

**Figure 4.**
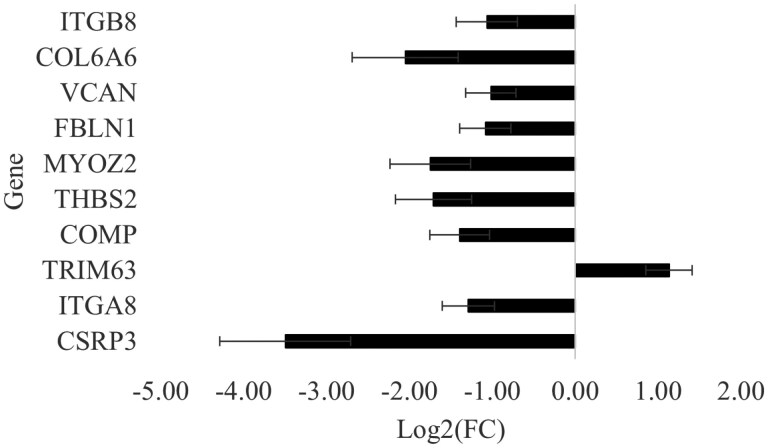
Log_2_(fold change) ± SE of significantly differentially expressed genes classed in the cytoskeleton in muscle cells pathway. *ITGA8* and *ITGB8*, Integrin subunit alpha and beta 8; *VCAN*, Versican; *COL6A6*, Collagen type VI alpha-6 chain; *THBS2*, Thrombospondin 2; *COMP*, Cartilage oligomeric matrix protein; *FBLN1*, Fibulin 1; *MYOZ2*, Myozenin 2; *TRIM63*, Tripartite motif containing 63; *CSRP3*, Cysteine and glycine rich protein 3.

A total of 10 DEGs were genes involved in the Cytoskeleton in muscle cell (CMC) pathway (Table 6). Except for the *TRIM63* gene, these genes were downregulated in the P. major muscle under the HS compared to the TN thermal condition (Figure 4). Four of these genes were also part of the ECM-receptor interaction pathway (*ITGA8*, *ITGB8*, *COL6A6*, *THBS2*), which is consistent with the role of the ECM as discussed above.

The Myozenin 2 (*MYOZ2*) gene encodes a protein associated with actin and myosin found in the Z-line of skeletal muscle, an essential component of the contractile apparatus (Takada et al., 2001). Human studies indicate that this gene is specific to slow-twitch and cardiac muscles, and a missense mutation in this gene is associated with hypertrophic cardiomyopathy (Osio et al., 2007). However, a previous study suggested that this gene was also involved in muscle fiber type regulation in the P. major muscle of broiler chickens (Bottje et al., 2017), which is composed of fast-twitch myofibers (type IIB).

The Tripartite Motif containing 63 (*TRIM63*, also known as *MuRF1*) gene encodes the Muscle-specific RING-Finger protein-1 (MuRF1), which ubiquitinates structural proteins and mediates their ubiquitin-proteasome system-dependent degradation during the process of muscle atrophy (Pablo Tortola et al., 2020). Chronic HS is known to increase the expression of *MuRF1* in the P. major muscle of broiler chickens (Ma et al., 2021a; Teyssier et al., 2023), which also contributes to muscle mass loss under HS conditions.

The Cysteine and Glycine Rich Protein-3 (*CSRP3*) gene encodes the LIM proteins that play a vital role in maintaining the structure and function of skeletal muscles (Cui et al., 2020). The silencing of this gene resulted in the downregulation of the expression of myogenic genes and the upregulation of atrophy-related genes in chicken myoblasts (Cui et al., 2020). Knockdown of *CSRP3* expression in satellite cells isolated from the P. major muscle reduces their proliferation and differentiation (Velleman et al., 2021). This gene also promotes the formation of autophagosomes and the activation of autophagy, a process essential for cell survival (Cui et al., 2020).

The *VCAN* gene encodes a large ECM proteoglycan called Versican (Wight et al., 2020). The G3 domain of Versican has 2 epidermal growth factor-like repeats that can enhance cell proliferation (Zhang et al., 1998). In a study on turkey breast muscles using a microarray, the expression of *VCAN* was found to be maximal at day 18 of embryogenesis, decreasing by 90% on the day of hatch, which suggests a role of this gene in muscle hyperplasia but not hypertrophy (Sporer et al., 2011). In a study using satellite cells isolated from turkey breast muscles, Velleman et al. (2012) found that knockdown of *VCAN* using small interfering RNAs (siRNAs) increased satellite cell proliferation at 24 h post-transfection, an effect that persisted through 72 h of proliferation. Yet, *VCAN* knockdown simultaneously decreased satellite cells differentiation, which suggests that Versican may play an important role in muscle cell differentiation into myoblasts that fuse together to form myotubes during myogenesis.

The *FBLN1* gene encodes for another glycoprotein of the ECM called Fibulin-1, involved in connective tissue proliferation in broiler breast muscles (Brothers et al., 2019). A previous study showed that stress conditions, such as high stocking density in broilers, led to the downregulation of this gene’s expression (Wu et al., 2020).

**Figure 5.**
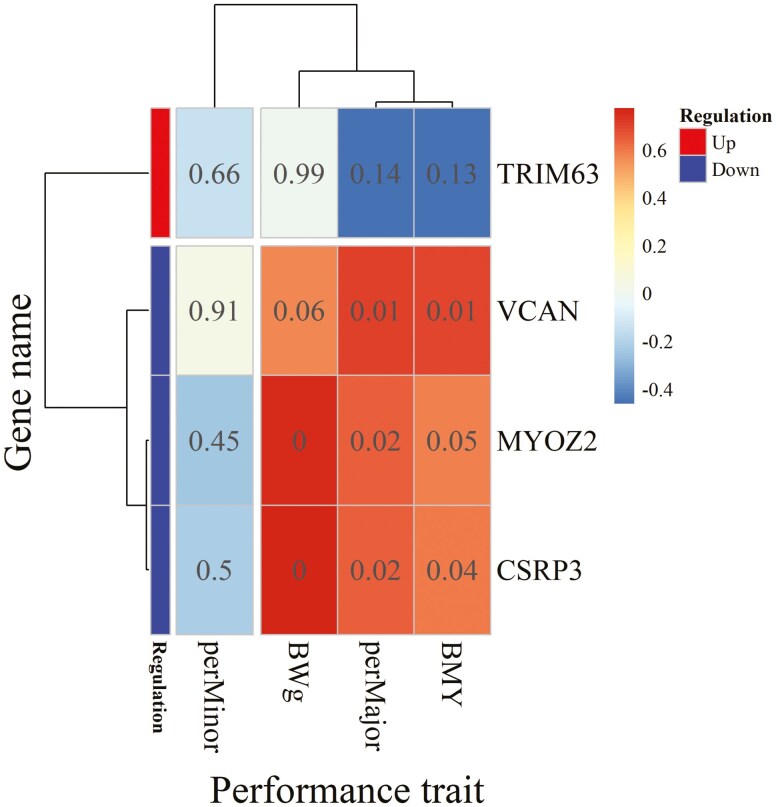
Heatmap of the correlations of significantly differentially expressed genes classed in the cytoskeleton in muscle cells pathway with body weight expressed in grams (BWg), yield of the whole breast (BMY), of the Pectoralis major (perMajor), and Pectoralis minor (perMinor) muscle. *TRIM63*, Tripartite motif containing 63; *CSRP3*, Cysteine and glycine rich protein 3; *VCAN*, Versican; *MYOZ2*, Myozenin 2. The color scale indicates the order of magnitude of the correlation coefficients, and numbers displayed in the cells of the heatmap are P-values of corresponding correlation coefficients.

Overall, our data suggest that HS is associated with the upregulation of genes involved in protein degradation and the downregulation of genes encoding structural proteins, which may contribute to impaired muscle cell structure and development. Figure 5 presents the correlations (*n* = 12 birds, 6 birds per group) between these 4 genes, BMY, muscle yield, and BW. BMY was positively correlated with *VCAN*, *MYOZ2*, and *CSRP3* expression, but not with *TRIM63*. Similar patterns were observed for P. major yield and BW, whereas P. minor yield did not correlate with any of these genes. These positive correlations, combined with the observed downregulation of the same genes under HS, are consistent with a potential role of this pathway in the reduced muscle growth and BW observed under HS conditions. However, as mentioned before, these associations require validation in larger studies and experimental designs that can separate the intrinsic effects of HS from those of reduced feed intake.

#### The regulation of the actin cytoskeleton pathway (KEGG: gga04810)

**Figure 6.**
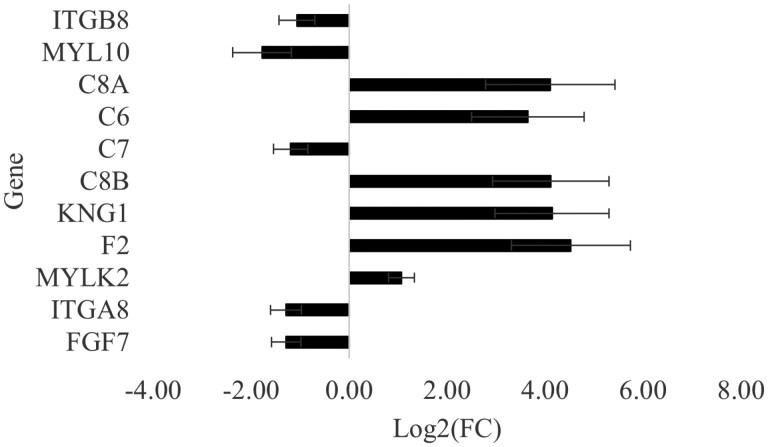
Log_2_(fold change) ± SE of significantly differentially expressed genes classed in the regulation of the actin cytoskeleton pathway. *ITGA8* and *ITGB8*, Integrin subunit alpha and beta 8, *C6*, *C7*, *C8A*, *C8B*, complement components 6, 7, 8A, and 8B, *MYLK2*, Myosin light chain kinase 2; *MYL10*, Myosin light chain 10; *KNG1*, Kininogen 1; *F2*, Coagulation factor II; *FGF7*, Fibroblast growth factor 7.

A total of 11 DEGs identified in the current study were classed in this pathway (Table 6). Two of these genes were also involved in the 2 previously discussed pathways (*ITGA8*, *ITGB8*). Of the remaining 9 genes, 6 were upregulated and 3 were downregulated (Figure 6). Interestingly, DAVID classed 4 genes encoding complement immune components (*C6*, *C7*, *C8A*, *C8B*) amongst genes contributing to this pathway. These genes were all upregulated except for *C7,* which was downregulated in the P. major muscle of the HS group. These components are part of the membrane attack complex, an effector of the innate and adaptive immunity (Xie et al., 2020). Upon muscle injury, a remodeling process is initiated as a structural adaptation in response to the injury. The accompanying inflammation activates the complement system that contributes to the recruitment of inflammatory cells to the injured region (Frenette et al., 2000). Studies utilizing in vitro (3D) and in vivo (mice) models showed that the activation of the complement system was required for efficient regeneration of injured tissue (Jacobs et al., 2018). Under HS thermal conditions, protein breakdown in the breast muscles, in conjunction with the infiltration of adipose tissue, triggers inflammation and oxidative damage (Ma et al., 2021a; Malila et al., 2022; Teyssier et al., 2023). In response, the complement system could be triggered in the muscle to recruit inflammatory cells and contribute to the above-discussed remodeling of HS-damaged muscle tissue. This statement is supported by the upregulation of the Kininogen 1 (*KNG1*) gene in our study, which has been previously associated with biological functions such as the activation of leukocytes and macrophages in broiler P. major muscle (Reyer et al., 2018).

Myosin light chain kinase 2 (*MYLK2*) gene was upregulated under the HS thermal condition compared to TN (Figure 6). The enzyme encoded by this gene plays a role in regulating muscle contraction by phosphorylating the regulatory light chain of myosin (Stull et al., 2011). Reduced levels of *MYLK2* in the P. major muscle of broilers were associated with low integrity of myofibrillar proteins and the development of the White striping phenotype (Marciano et al., 2021). Given the damaging effect of HS on breast muscles, it could be postulated that the upregulation of this gene is potentially a compensatory response to the compromised muscle integrity under HS. Conversely, the myosin regulatory light chain 10 (*MYL10*) gene was downregulated in the P. major muscle of the HS group. In a study comparing the transcriptomic profiles of 2 broiler lines selected for high or low feed efficiency, the expression of *MYL10* was found to be downregulated in the P. major muscle of the high feed efficiency line compared to the low feed efficiency line (Kong et al., 2011). These authors suggested that the downregulation of this gene may reduce energy expenditure and increase energy efficiency because the cytoskeletal functions it supports are highly energy-demanding. Based on this, it is suggested that energy metabolism in the P. major muscle is disrupted under HS thermal conditions. Consequently, the downregulation of this gene in our study could be a part of an adaptive response to enhance energy efficiency under HS thermal conditions, which is supported by the absence of difference in glycolytic metabolites between the 2 thermal conditions in our study.

The Fibroblast growth factor 7 (*FGF7*) gene was also downregulated in the P. major muscle under the HS thermal condition. This gene is mainly expressed in fibroblast cells and specifically affects epithelial cells (Zheng et al., 2024). In a study using porcine satellite cells and single-cell RNA sequencing to elucidate the interaction between fibro-adipogenic progenitors and satellite cells, *FGF7* was found to mediate this interaction, and exogenous FGF7 supplementation was found to increase the proliferation of satellite cells, benefiting muscle development (Ma et al., 2024). The downregulation of this gene by HS is therefore consistent with the detrimental effect of HS on muscle growth.

**Figure 7.**
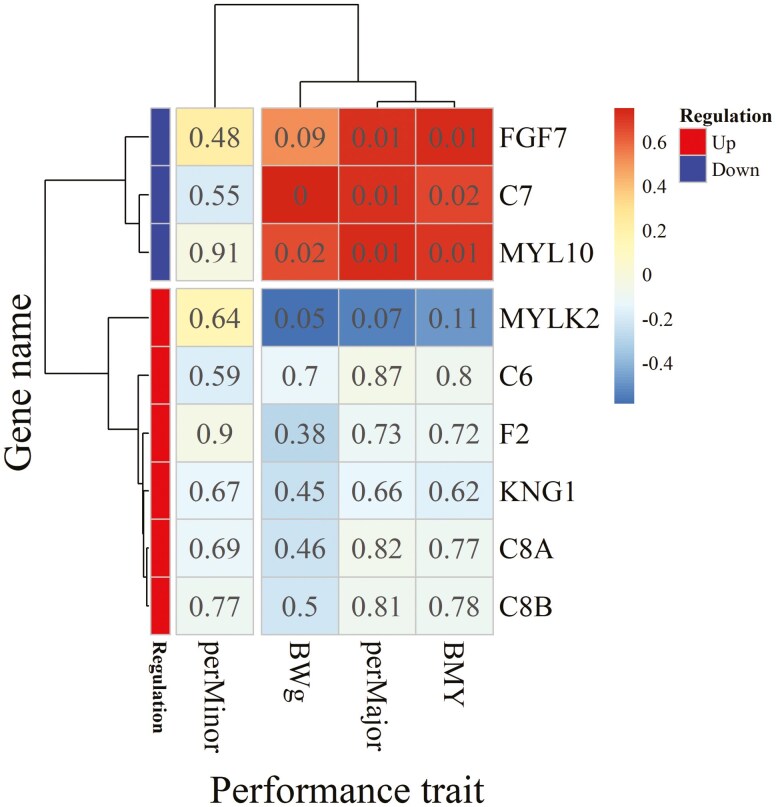
Heatmap of the correlations of significantly differentially expressed genes classed in the regulation of actin cytoskeleton pathway with body weight expressed in grams (BWg), yield of the whole breast (BMY), of the Pectoralis major (perMajor), and Pectoralis minor (perMinor) muscle. *C6*, *C7*, *C8A*, *C8B*, complement components 6, 7, 8A, and 8B; *MYLK2*, Myosin light chain kinase 2; *MYL10*, Myosin light chain 10; *KNG1*, Kininogen 1; *F2*, Coagulation factor II; *FGF7*, Fibroblast growth factor 7. The color scale indicates the order of magnitude of the correlation coefficients, and numbers displayed in the cells of the heatmap are P-values of corresponding correlation coefficients.

Figure 7 illustrates the correlations (*n* = 12 birds, 6 birds per group) between genes classified in the regulation of the actin cytoskeleton pathway and traits related to muscle mass and BW. Three genes (*C7*, *FGF7*, and *MYL10*) were positively correlated with P. major yield, BMY, and BW, consistent with the functional roles previously attributed to these genes. Notably, all 3 were downregulated under HS conditions. These findings suggest a potential link between the downregulation of actin cytoskeleton-related genes and reduced muscle growth under HS. However, given the absence of a pair-fed group in our study, these associations require validation in larger studies with pair-feeding designs.

### Limitations

This study did not include a pair-fed group, which prevents us from separating the direct effects of HS from those mediated by reduced feed intake. To further explore the relationship between gene expression and performance traits, we conducted correlation analyses between normalized transcript counts of identified DEGs and growth-related traits (BW, BMY, P. major, and P. minor yields). Although several correlations were statistically significant, these analyses were based on a relatively small sample size (*n* = 12, 6 birds per treatment) and should therefore be considered exploratory. The results provide preliminary evidence of a potential link between genes classed in the above-discussed pathways and muscle yield, but they require confirmation in future studies with larger sample sizes and experimental designs (e.g., pair-feeding) that can isolate the intrinsic effects of HS from those of reduced feed intake.

## Conclusions

In the present study, we investigated the effects of chronic cyclic HS of mild intensity on growth, breast meat yield, meat quality, glycolytic metabolites, and gene expression in the Pectoralis major muscle of broiler chickens. The compensatory feed intake between stress cycles did not reduce the lasting effect of HS on BW and BMY. In terms of meat quality, our study confirmed findings from the literature by showing that chronic cycle HS has a very limited to no effect on quality traits, potentially due to short-term feed intake compensation between stress cycles, allowing the reconstitution of muscle glycolytic reserves. Original findings from this study using transcriptomics showed that HS downregulated genes that are positively correlated with BW and meat yield. These genes have functional roles in muscle growth, repair, and inflammatory responses, which led to the decreases in BW and meat yield. More specifically, genes involved in the extracellular matrix-interaction pathway (KEGG: gga04512), the CMCs pathway (KEGG: gga04820), and the regulation of actin cytoskeleton pathway (KEGG: gga04810), which are mostly positively correlated with muscle mass and BW, were found to be downregulated by HS. Given the important role of these pathways in muscle growth, structural integrity, and repair, their downregulation by HS partly explains the loss of BW and muscle mass under HS. While these findings are descriptive due to the absence of a pair-fed group, they identify candidate pathways potentially involved in HS responses. Future studies including pair-fed controls will be necessary to separate the intrinsic effects of HS from those of reduced feed intake.

## Supplementary Material

skaf310_suppl_Supplementary_Materials_1

## Data Availability

The data underlying this article are available in the article and in its online supplementary material.

## Abbreviations

a*: redness index of the Pectoralis major muscle
b*: yellowness index of the Pectoralis major muscle
BW: body weight
BMY: breast meat yield
CL: cooking loss
CL2: cooking loss after freezing, thawing, marination and cooking
CMC: Cytoskeleton in muscle cell
DL: drip loss
DEGs: differentially expressed genes
EAI: the emulsion activity index
ECM: extracellular matrix
ESI: the emulsion stability index
FTL: freezing-thawing loss of breast meat
Glucose-6-P: glucose-6-phosphate
HS: heat stress
L*: lightness of the Pectoralis major muscle
P. Major: the Pectoralis major muscle
P. Minor: the Pectoralis minor muscle
pH: breast muscle pH
pHu: the ultimate pH of the Pectoralis major muscle
pHi: the initial pH of the Pectoralis major muscle
MORS: Meullenet-Owens razor shear force
TN: thermoneutral
WBSF: Warner-Bratzler shear force of breast meat
